# A case series of negative pressure wound therapy as a promising treatment in patients with burn injury

**DOI:** 10.1016/j.ijscr.2020.03.034

**Published:** 2020-04-01

**Authors:** M. Rosadi Seswandhana, Sharfan Anzhari, Ishandono Dachlan, Yohanes Widodo Wirohadidjojo, Teguh Aryandono

**Affiliations:** a Division of Plastic, Reconstructive, and Aesthetic Surgery, Department of Surgery, Dr. Sardjito Hospital/Faculty of Medicine, Public Health, and Nursing, Universitas Gadjah Mada, Yogyakarta, Indonesia; b Faculty of Medicine, Public Health, and Nursing, Universitas Gadjah Mada, Yogyakarta, Indonesia; c Department of Dermatovenereology, Dr. Sardjito Hospital/Faculty of Medicine, Public Health, and Nursing, Universitas Gadjah Mada, Yogyakarta, Indonesia; d Division of Surgical Oncology, Department of Surgery, Dr. Sardjito Hospital/Faculty of Medicine, Public Health, and Nursing, Universitas Gadjah Mada, Yogyakarta, Indonesia

**Keywords:** Burns, Burn dressing, Burn treatment, Negative pressure wound therapy, NPWT

## Abstract

**Introduction:**

Burn injuries continue to be a global burden. Burn wound treatment often requires a long treatment time and often results in high morbidity and mortality. Until now, there is still no ideal burn wound treatment for all patients.

**Presentation of cases:**

Here we report 3 cases of burn patients. Every patient underwent wound debridement and followed by installation of Negative Pressure Wound Therapy (NPWT) on the site of burn injuries. All of patients was discharged in a good condition.

**Discussion:**

NPWT is an application of sub-atmospheric pressure that is placed on the wound. Today, the use of negative pressure therapy is a new strategy in managing burn injuries. NPWT can reduce the length of stay of burn patients that can be associated with faster wound healing, and reduce bacterial levels in burns.

**Conclusion:**

The combination of debridement and NPWT in burn patients can be an alternative procedure because it can promote faster wound healing and reduce the length of hospitalization. Furthermore, this is the first report of NPWT usage for the treatment of burn patients in Indonesia.

## Highlights

• NPWT significantly reduced length of hospitalization.
• Also had minimal complication.
• This procedure can be an alternative treatment for burn patients.

## 1 Introduction

Burn injuries have become a major global health problem with fire-related burn injuries causing an estimated 195,000 deaths annually [1]. Burn injuries are also a leading cause of mortality and morbidity in low and middle-income countries, with the Southeast Asian region accounting for 59% of burns deaths. The latest data on burns in Indonesia from the Ministry of Health published in 2014 shows that burns rank 6th on the list of accidental injuries [2]. Problems for the patients related to burn injuries include not only potential for fatality from complications such as infection and sepsis but also they are a financial burden to the public healthcare system because of the need for increased resources during the extended treatment period [3,4].

According to the ISBI Practice Guideline from 2016, there is still no ideal dressing that would be adapted to burn wounds at all times. The ideal dressing should be able to prevent contamination, dryness, evaporation, and have minimal adverse effects. The dressing is expected to be able to be left attached to the wound surface for an extended time so it can provide better wound healing outcomes [5]. Negative pressure wound therapy (NPWT) is a dressing method that has been widely used and has become a standard therapy in several cases of surgical wound treatment. NPWT is considered able to provide an optimal wound healing environment, promote re-epithelialization, reduce edema and bacterial load, and increase dermal perfusion rate [6,7]. However, further studies regarding the benefits of NPWT in burns are still minimally available.

This study aimed to show the results of burn wound treatment using NPWT. The research has been reported in line with the PROCESS criteria [8]

## 2 Case report

### 2.1 Case 1

**Fig. 1:**
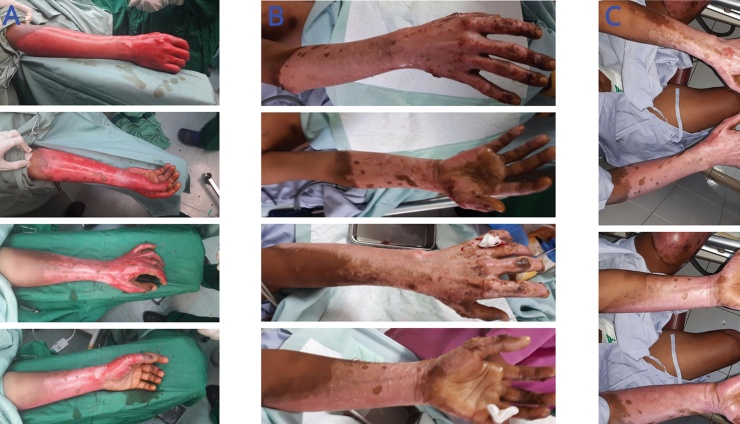
A) The first-day patient was admitted to the hospital, burns on the right forearm were combination of pale pink, dark pink, and blotchy red indicating the depth of the superficial to deep dermal burns. B) 10th day of admission, after installation of NPWT for 5 days. C) 15th day of admission, after installation of NPWT for 10 days.

Male, 23 years old, was referred to the emergency department because of fire-related burn injury. The patient suffered from superficial to deep dermal burn injuries that covered 22% of the total body surface area (TBSA). The patient was admitted to the burn unit department. On the 5th day of admission, the patient underwent the wound debridement and installation of NPWT. Burn wounds were cleaned every five days, followed by re-installation of NPWT. On the 15th day of admission, the wound was managed with open wound treatment using Vaseline without installation of NPWT. The patient was discharged on the 19th day of treatment in a good condition (Fig. 1).

### 2.2 Case 2

**Fig. 2:**
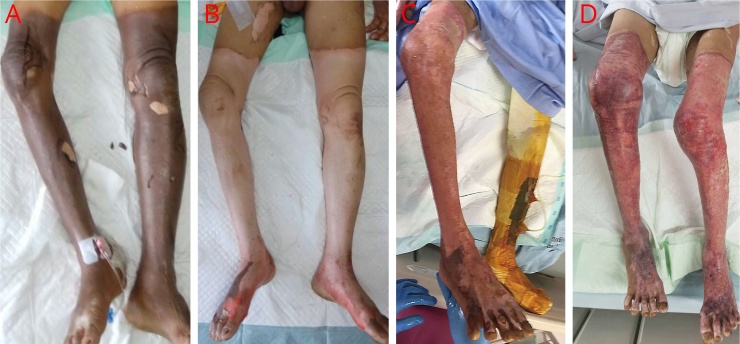
A) The first-day patient was admitted to the hospital, burns were seen on both patient’s lower limbs. B) Post wound debridement. C) 20th day of admission showed the re-installation of NPWT. Note that the left lower leg was mounted with NPWT dressings. D) 30th day of admission, NPWT installation has been completed. Patient care was then continued with open wound treatment using Vaseline.

Male, 56 years old, was referred to the emergency department because of fire-related burn injury. The patient suffered from superficial to deep dermal burn injuries that covered 53% TBSA. On the 10th day of admission, the patient underwent the wound debridement and installation of NPWT. Burn wound was cleaned every five days, followed by re-installation of NPWT. On the 31st day of admission, the burn wound surfaces were decreased by 48% TBSA. The patient should be discharged that day, but due to social factors, the patient still being treated at the hospital with open wound care using Vaseline until the 51st day of admission before being discharged in a good condition (Fig. 2).

### 2.3 Case 3

**Fig. 3:**
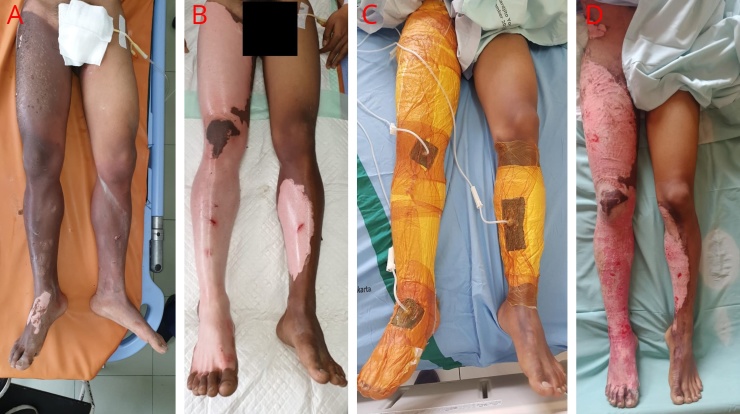
A) The first-day patient was admitted to the hospital, burns were seen on the entire right lower leg and left calf. B) Post wound debridement. C) Installation of NPWT after wound debridement. D) 10th day of admission, post NPWT installation.

Male, 19 years old, was admitted to the emergency department because of electrical-related burn injury. The patient suffered from superficial to mid dermal injuries that cover 21% TBSA. On the 5th day of admission, the patient underwent the wound debridement and installation of NPWT. On the 19th day of admission, the burn wound surface reduced about 20% TBSA. The patient was discharged on the 21st day of treatment in a good condition (Fig. 3).

NPWT Procedure:

1. Clean the burn wound using normal saline.
2. Cover the burn wound with three layers of sterile gauzes until wrapping all of the wound surfaces.
3. Put the connector cup at the center of the wound.
4. Cover the gauzes with an occlusive transparent film dressing.
5. Connect the connector cup with the hose which was already connected to the NPWT device with a pressure setting of −125 mmHg.
6. Evaluate the wound every 5 days.

## 3 Discussion

The main approach to burn treatment is very dependent on the degree of burn injury. It is expected to reduce pain, prevent infection, promote healing, with long-term effects to minimize scar tissue and contractures [9]. NPWT is an application of sub-atmospheric pressure that is placed on the wound. Today, the use of negative pressure therapy is a new strategy in managing burn injuries [6,10].

One of the problems with burn care is the extended length of stay in the hospital. In this study, the average length of stay was 30.33 (19.00–51.00) days. Every 1% of TBSA is ideally equivalent to 1 day of hospitalization. However, several factors can affect the length of stay such as gender, age, TBSA, depth of the wound, infection/sepsis, location of burns, and inhalation injury [3]. In our cases, there was faster recovery based on TBSA. There was a decrease in length of stay in all cases by 13.6%, 3.7%, and 9.5%, respectively. According to a recent study, NPWT can reduce 78% of the length of stay of burn patients [10]. Shortening of length of stay is also associated with faster wound healing. Recently, there have been many studies related to the use of stem cells in wound healing. Burns gives rise to several potential indications for the application of stem cells such as expediting wound healing, improving skin regeneration, and reducing fibrosis [11]. The use of NPWT alone can increase differentiation from mesenchymal stem cells (MSC) thereby increasing cellular proliferation which can help accelerate wound healing [12]. These findings are in line with a study conducted by Yang et al. [13] which stated that a combination of NPWT and potential stem cell therapy can be a safer and more effective method for tissue growth in burn patients compared to conventional methods. Other evidence shows that NPWT can be combined with skin graft in burn patients. Negative pressure dressing can improve graft take, speed up the process, and reduce the duration of graft dressings compared to conventional dressing covered with Vaseline gauze alone [14]. This evidence indicates that the use of NPWT is very effective in patients with superficial to deep-dermal burn degree with or without using any additional skin graft therapy.

At the end of the study, all patients were discharged from hospital in good condition. Another study in Indonesia showed around 21% of burn patients treated with 10–60% TBSA die [3]. This finding shows that the use of NPWT can help reduce the mortality rate of patients who are hospitalized. Burn sepsis is one of the leading causes of mortality and morbidity of burn patients treated in hospitals [3,10]. In previous studies, the use of NPWT was considered able to reduce bacterial levels in burns. According to Ibrahim et al. [15], there was a significant decrease in bacterial colonization on the 21st day of treatment using NPWT compared to standard therapy. Another study conducted by Kement and Baskiran [16] stated that no bacterial growth was found in burn tissue culture that was given NPWT 100–150 mmHg continuously for 72 h. This indicates that NPWT can be an excellent therapeutic choice for burn patients.

A Cochrane study published in 2014 stated that the effectiveness and safety of NPWT usage in burns, especially partial-thickness burns, could not be concluded due to a lack of data [17]. The latest Cochrane study published in 2019 also concluded that there was still uncertainty about the effect of the usage of NPWT on healing and wound complications when compared to standard dressing [18]. This finding is contrary to the results of this study where burn patients who received NPWT therapy had good outcomes, especially in terms of shorter length of stay and no mortality.

## 4 Conclusion

NPWT can be considered as a new dressing therapy for burn patients. At the end of the study, patients were discharged in good condition, and none of the patients died. However, we recommend conducting a trial study involving more burn patients and the usage of standard NPWT technique.

## Declaration of Competing Interest

No potential conflict of interest relevant to this article was reported.

## Funding

The authors declare that this study had no funding resource.

## Ethical approval

The informed consent form was declared that patient data or samples will be used for educational or research purposes. Our institutional review board also do not provide an ethical approval in the form of case series.

## Consent

Written informed consent was obtained from the patients for publication of this case report and accompanying images. A copy of the written consent is available for review by the Editor-in-Chief of this journal on request.

## Author contribution

M Rosadi Seswandhana conceived the study. Sharfan Anzhari drafted the manuscript. Ishandono Dachlan, Yohanes Widodo Wirohadidjojo, and Teguh Aryandono critically revised the manuscript for important intellectual content.

## Registration of research studies

ClinicalTrials.gov Identifier: NCT00548314.

## Guarantor

M Rosadi Seswandhana.

## Provenance and peer review

Editorially reviewed, not externally peer-reviewed.
